# Neural activity changes in first-episode, drug-naïve patients with major depressive disorder after transcutaneous auricular vagus nerve stimulation treatment: A resting-state fMRI study

**DOI:** 10.3389/fnins.2022.1018387

**Published:** 2022-10-12

**Authors:** Sijie Yi, Zhi Wang, Wenhan Yang, Chuxin Huang, Ping Liu, Yanjing Chen, Huiting Zhang, Guangju Zhao, Weihui Li, Jiliang Fang, Jun Liu

**Affiliations:** ^1^Department of Radiology, The Second Xiangya Hospital of Central South University, Changsha, China; ^2^Department of Radiology, Guang’anmen Hospital, China Academy of Chinese Medical Sciences, Beijing, China; ^3^MR Scientific Marketing, Siemens Healthineers Ltd., Wuhan, China; ^4^Department of Psychiatry, National Clinical Research Center for Mental Disorders, The Second Xiangya Hospital of Central South University, Changsha, China; ^5^Clinical Research Center for Medical Imaging in Hunan Province, Changsha, China; ^6^Department of Radiology Quality Control Center, Changsha, China

**Keywords:** major depressive disorder, first-episode, drug-naïve, treatment, regional homogeneity, transcutaneous auricular vagus nerve stimulation, resting-state fMRI

## Abstract

**Introduction:**

Major depressive disorder (MDD) is a disease with prominent individual, medical, and economic impacts. Drug therapy and other treatment methods (such as Electroconvulsive therapy) may induce treatment-resistance and have associated side effects including loss of memory, decrease of reaction time, and residual symptoms. Transcutaneous auricular vagus nerve stimulation (taVNS) is a novel and non-invasive treatment approach which stimulates brain structures with no side-effects. However, it remains little understood whether and how the neural activation is modulated by taVNS in MDD patients. Herein, we used the regional homogeneity (ReHo) to investigate the brain activity in first-episode, drug-naïve MDD patients after taVNS treatment.

**Materials and methods:**

Twenty-two first-episode, drug-naïve MDD patients were enrolled in the study. These patients received the first taVNS treatment at the baseline time, and underwent resting-state MRI scanning twice, before and after taVNS. All the patients then received taVNS treatments for 4 weeks. The severity of depression was assessed by the 17-item Hamilton Depression Rating Scale (HAMD) at the baseline time and after 4-week’s treatment. Pearson analysis was used to assess the correlation between alterations of ReHo and changes of the HAMD scores. Two patients were excluded due to excessive head movement, two patients lack clinical data in the fourth week, thus, imaging analysis was performed in 20 patients, while correlation analysis between clinical and imaging data was performed in only 18 patients.

**Results:**

There were significant differences in the ReHo values in first-episode, drug-naïve MDD patients between pre- or post- taVNS. The primary finding is that the patients exhibited a significantly lower ReHo in the left/right median cingulate cortex, the left precentral gyrus, the left postcentral gyrus, the right calcarine cortex, the left supplementary motor area, the left paracentral lobule, and the right lingual gyrus. Pearson analysis revealed a positive correlation between changes of ReHo in the right median cingulate cortex/the left supplementary motor area and changes of HAMD scores after taVNS.

**Conclusion:**

The decreased ReHo were found after taVNS. The sensorimotor, limbic and visual-related brain regions may play an important role in understanding the underlying neural mechanisms and be the target brain regions in the further therapy.

## Introduction

Major depressive disorder (MDD) is a disease with prominent individual, medical, and economic impacts, characterized by pessimism, social isolation, low self-confidence and mental inhibition (Kessler et al., 2010; Stegmann et al., 2010). Drug therapy is currently the most common treatment for MDD, although it may induce drug-resistance (Miller et al., 2022) and have associated side effects such as loss of memory, decrease of reaction time, and residual symptoms. Other treatments on MDD patients such as electroconvulsive therapy (ECT) are mostly invasive and may have adverse cognitive effects and residual functional impairments (Wang et al., 2020). One-third of MDD patients do not respond to traditional treatments such as antidepressants, psychotherapy, or cognitive therapies (Fava, 2003). As a result, it is clinically urgent to develop a new effective treatment strategy for MDD.

Vagus nerve stimulation (VNS) has been approved as a treatment for treatment-resistant MDD (Daban et al., 2008). VNS has superior clinical results and higher remission rates in MDD treatment (Schlaepfer et al., 2008; Aaronson et al., 2017). However, as an invasive procedure, the technical challenges, surgical risks, and potential side effects have all restricted the wide use of VNS in clinical practice (Fitzgerald, 2013). A non-invasive way that stimulates the vagus afferent would be an important advancement in MDD treatment.

Transcutaneous auricular vagus nerve stimulation (taVNS) has been developed as a new method for non-invasively stimulating brain regions in a similar fashion to VNS in order to reduce depressive symptoms (Ventureyra, 2000; Fallgatter et al., 2005). The pattern of brain regions modulated by taVNS in MDD patients is similar to that obtained with VNS (Frangos et al., 2015; Fang et al., 2016). Some studies have shown that taVNS treatment can modulate the brain function of MDD patients and significantly improve depressive symptoms (Fang et al., 2016, 2017; Liu et al., 2016). However, The MDD patients in these investigations were treated with medication, which could affect changes in brain functions. The taVNS treatment has the advantages of being safe and non-invasive, and with no negative effects (Kraus et al., 2007).

These preliminary investigations have revealed that taVNS treatment has promising results in the treatment of MDD patients, but the underlying mechanisms need to be explored further. Regional homogeneity (ReHo) reveals the internal function of the brain and is linked to external behavioral manifestations. Previous research has suggested that abnormal ReHo could be the result of localized brain function imbalance or a decompensation reaction involving the entire brain network (Dai et al., 2012; Zuo et al., 2013). Multiple studies have shown that ReHo values in the precentral gyrus (PrCG) (Liu et al., 2021), the postcentral gyrus (PoCG) (Xia et al., 2019; Liu et al., 2021), the calcarine cortex (CAL) (Shen et al., 2017), the supplementary motor area (SMA) (Yan et al., 2021), and the lingual gyrus (LG) (Geng et al., 2019) are all increased in MDD patients compared with healthy controls (HCs).

The purpose of this study was to explore whether and how taVNS treatment modulated ReHo values in first-episode, drug-naïve MDD patients. Twenty-two MDD patients were given their first taVNS therapy at the baseline time, and resting-state MRI scanning was performed twice before and after taVNS. They then underwent a 4-week taVNS treatment. At the baseline and the 4-week follow-up, the 17-item Hamilton Depression Rating Scale (HAMD) (Hamilton, 1967) was used to determine the MDD severity. Before and after taVNS, paired *t*-tests were performed in MDD patients to assess ReHo alterations. Furthermore, Pearson correlation analyses were used to investigate the relationship between changes in ReHo values and clinical improvements in MDD patients before and after taVNS. We hypothesized that taVNS treatment of MDD patients significantly modulated ReHo values in some brain regions, and that changes in ReHo values in these brain regions may be perceived as the effective taVNS treatment of MDD patients in the underlying neural mechanisms.

## Materials and methods

### Subjects

This is a longitudinal research project. Twenty-two right-handed MDD patients, aged from 18 to 51, were recruited from the Mental Health Institute of Central South University’s Second Xiangya Hospital. Two certified psychiatrists used the Structured Clinical Interview to diagnose major depression using DSM-IV criteria. All the patients were experiencing their first episode of MDD and were untreated. This study enrolled patients who voluntarily provided informed consent and met the inclusion criteria. The 17-item HAMD was used to determine the severity of MDD. The total score of the 17-item HAMD was counted as 17 or more in all the subjects. Bipolar disorder, organic mental disorder, drug-induced depression, seasonal affective disorder, major sickness, pregnancy, postpartum depression, dementia or other cognitive impairment were taken as the exclusion criteria. Patients who refused to sign the consent form were also excluded.

### Procedures

The purpose of the clinical experiment was to investigate the antidepressant effects of taVNS treatment. During the timespan of 4 weeks, all the patients got taVNS treatment. The fMRI scan was applied to the resting state before taVNS treatment, and another fMRI scan was applied in the same fashion 30 min after taVNS treatment.

### Intervention

The afferent branch of the vagus nerve on the surface of the ear is stimulated by taVNS, which is the sole area with vagus nerve distribution on the body surface (Rong et al., 2012; Hein et al., 2013). The stimulation locations for taVNS are in the bilateral auricular concha area that has a dense dispersion of vagus nerve branches. Electrodes were applied to the ear area at the stimulation site after we cleaned the bilateral auricle area.

The followings were the stimulation parameters: (1) density wave set to 20 Hz with wave width smaller than 1 millisecond, and (2) intensity adjusted to the patient’s tolerance (typically between 4 and 6 mA). Each treatment lasted 30 min and was given twice a day (once in the morning and once in the evening) for at least 5 days a week during the treatment period (4 weeks).

All subsequent treatments were self-administered by the patients at home after proper instruction following the baseline MRI scan. Patients were also advised to fill out a patient diary booklet each day to record any side effects that were related to or coincided with therapy. The patient diaries were examined by the investigators at the end of the 4-week treatment.

### Clinical outcomes

The 17-item Hamilton Depression Rating Scale was the primary clinical outcome measure in this study, with secondary outcome measures including the 17-item Hamilton Anxiety Rating Scale (HAMA), the Self-Rating Anxiety Scale (SAS), and the Self-Rating Depression Scale (SDS) measured at the baseline time and in 4-week follow-up.

### Data acquisition

A 3.0 T MRI equipment (MAGNETOM Skyra, Siemens Healthcare, Germany) with a standard 20-channel combination head and neck coil was used to collect fMRI data. The subjects were instructed to stay awake, close their eyes, keep their heads motionless, and think about nothing during the scan. To rule out organic brain lesions, we used simple T2-weighted imaging MRI. T1-weighted high-resolution structural images were collected prior to the functional run using the three-dimensional fast spoiled gradient-echo sequence (echo time 2.07 ms, repetition time 2530 ms, matrix 256 × 256, field of view 256 mm × 256 mm, flip angle 7°, voxel size 1.0 mm × 1.0 mm × 0.9 mm, slice thickness 0.9 mm, gap 0.45 mm, 192 slices). The functional data was then collected using a blood oxygen level dependent sequence. The gradient echo echo-planar imaging sequence (echo time 30 ms, repetition time 2000 ms, matrix 64 × 64, field of view 224 mm × 224 mm, flip angle 90°, voxel size 3.5 mm × 3.5 mm × 3.5 mm, slice thickness 3.5 mm, gap 0.875 mm, 32 slices, parallel to the anterior commissure-posterior commissure line) was used to acquire T2-weighted functional images encompassing the entire brain. Before taVNS treatment, a resting-state fMRI scan was performed. After 30 min of continuous stimulations of the bilateral auricular area, the resting fMRI scan was done again.

### fMRI data preprocessing

DPARSF V5.2^1^ based on the MATLAB platform was used to preprocess and analyze the fMRI data (Version No. R2016a). The procedures were as follows: The data from 10 time points were discarded when the DICOM format was transformed. The head motion and acquisition delay between slices were adjusted in the fMRI pictures. The values for translation (mm) and rotation (degrees) for each subject were estimated (the individuals should have no more than 2 mm maximum displacement in x, y, or z and no more than 2° of angular motion for the entire fMRI scan). After slice acquisition correction and head motion correction, the fMRI images were resampled to 3 mm × 3 mm × 3 mm and registered to the standard space. For subsequent ReHo analysis, the resultant fMRI data were temporally bandpass filtered (0.01–0.08 Hz) to decrease low-frequency drift and physiological high-frequency respiratory and cardiac noise, as well as time series linear detrending. The significance level of the statistical map that resulted was set to *p* < 0.05 (FWE corrected).

### Statistical analyses

#### Clinical data analyses

SPSS 25 was used for all statistical analyses of clinical ratings (IBM SPSS Statistics for Windows, Version 25.0. Armonk, NY: IBM Corp.). The treatment effects (baseline vs. week 4) in first-episode MDD patients were compared using a paired *t*-test.

#### fMRI data analyses

The changes in ReHo values before and after taVNS treatments were calculated using a paired *t*-test. Multiple comparisons were corrected using a threshold of a cluster-level *p* < 0.05 (FWE corrected).

#### Correlations

We calculated the improvement in HAMD scores after 4 weeks of taVNS treatment. In addition, pre-treatment and post-treatment ReHo values were extracted from resting state fMRI scans, and changes in ReHo values (post-treatment vs. pre-treatment) were calculated. Correlation between alterations of ReHo and changes of the HAMD scores were investigated using Pearson analysis.

## Results

### Clinical outcome

The baseline MRI scan was performed on the 22 patients across the entire research. Due to head motions, data from two patients were removed. At the end of week 4, two patient were removed from the research due to loss of follow-up. Of the 20 MDD patients who completed baseline resting state fMRI scans, only 18 received all the tests. Table 1 shows the patients’ demographics as well as the severity of their diseases.

**TABLE 1:** Demographic information and disease severity for MDD patients.

Demographic data	MDD patients (*n* = 20)
Gender (male/female)	5/15
Age (years)	27.70 ± 8.48
Years of education (years)	15.05 ± 2.06
HAMD score	20.10 ± 3.09
HAMA score	20.20 ± 7.89
SDS score	51.95 ± 6.56
SAS score	43.25 ± 7.60

Unless otherwise indicated values shown are mean ± SD. MDD, major depressive disorder; HAMD, Hamilton Depression Rating Scale; HAMA, Hamilton Anxiety Rating Scale; SAS, Self-Rating Anxiety Scale; SDS, Self-Rating Depression Scale.

Table 2 includes a summary of post-treatment clinical outcomes. Overall, the effects of taVNS treatment (pre-treatment vs. post-treatment) were substantial in all measurements. The scores from HAMD, HAMA, SDS, and SAS (HAMD: *t* = 8.756, *p* < 0.0001; HAMA: *t* = 4.916, *p* = 0.0001; SDS: *t* = 3.907, *p* = 0.0011; SAS: *t* = 5.823, *p* < 0.0001) all showed significant decline.

**TABLE 2:** Clinical outcome in MDD patients (pre-treatment and post-treatment).

Clinical outcome	MDD patients (*n* = 18)		Effect of treatment
		
	Pre-treatment	Post-treatment	
HAMD score	20.11 ± 3.10	9.83 ± 4.03	*t* = 8.756, *p* < 0.0001
HAMA score	20.83 ± 7.93	10.67 ± 5.38	t = 4.916, *p* = 0.0001
SDS score	52.50 ± 6.25	44.56 ± 7.81	t = 3.907, *p* = 0.0011
SAS score	43.83 ± 7.17	37.50 ± 7.17	t = 5.823, *p* < 0.0001

Unless otherwise indicated values shown are mean ± SD. MDD, major depressive disorder; HAMD, Hamilton Depression Rating Scale; HAMA, Hamilton Anxiety Rating Scale; SAS, Self-Rating Anxiety Scale; SDS, Self-Rating Depression Scale.

### Regional homogeneity results

**FIGURE 1:**
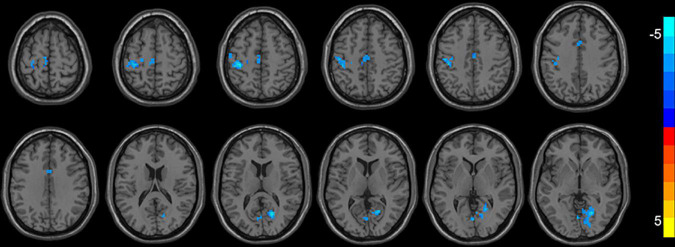
Brain regions with decreased ReHo (FWE corrected) in the first-episode MDD patients after taVNS are superimposed on a T1W template (first-episode MDD patients before and after treatment, paired-sample *T*-test). These regions included the left/right median cingulate cortex, the left precentral gyrus, the left postcentral gyrus, the right calcarine, the left supplementary motor area, the left paracentral lobule, and the right lingual gyrus with decreased ReHo after taVNS. The color bar signifies the *T*-value of the group analysis.

As shown in Table 3 and Figure 1, compared with ReHo values before treatments, ReHo values in MDD patients after treatments were significantly reduced in the left/right median cingulate cortex (MCC), the left precentral gyrus (PrCG), the left postcentral gyrus (PoCG), the right calcarine cortex (CAL), the left supplementary motor area (SMA), the left paracentral lobule (PAL), and the right lingual gyrus (LG) (*p* < 0.05, corrected). No significantly higher ReHo values were found in the whole brain.

**TABLE 3:** Brain regions with differences in ReHo values before and after stimulations in MDD patients.

Brain regions with lower ReHo value in the MDD patients	Peak MNI coordinate			T	Voxel size
			
	X	Y	Z		
**Decreased ReHo values**					
The left/right median cingulate cortex	0	9	36	–4.859	25
The left precentral gyrus	–36	–30	54	–5.208	35
The left postcentral gyrus	–36	–30	54	–5.208	120
The right calcarine fissure	21	–51	–15	–5.8447	66
The right lingual gyrus	21	–51	–15	–5.8447	120
The left supplementary motor area	–3	–21	54	–3.886	29
The left paracentral lobule	–3	–21	54	–3.886	27

MNI, Montreal Neurological Institute; MDD, major depressive disorder. *p* < 0.05 (FWE corrected).

### Correlations

**FIGURE 2:**
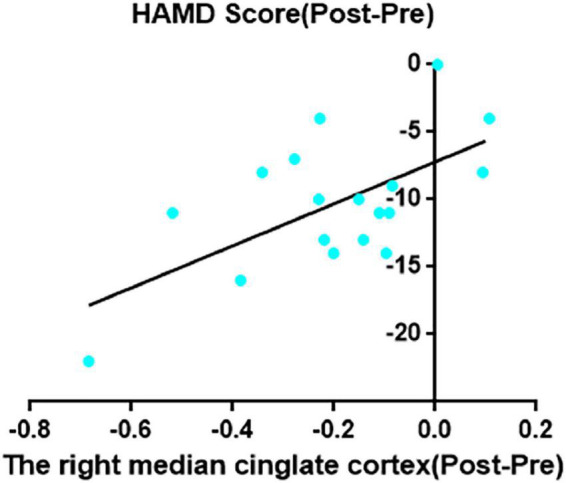
The right median cingulate cortex decreased ReHo after taVNS was significantly correlated with HAMD scores improvements (*r* = 0.62, *p* = 0.006, uncorrected).

**FIGURE 3:**
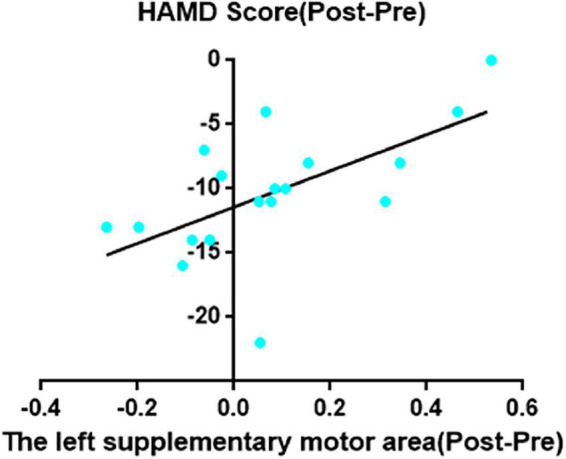
The left supplementary motor area decreased ReHo after taVNS was significantly correlated with HAMD scores improvements (*r* = 0.74, *p* = 0.0005, uncorrected).

Decreased ReHo in right MCC after taVNS was significantly correlated with HAMD improvements (*r* = 0.62, *p* = 0.006; Figure 2). In addition, the left SMA decreased ReHo after taVNS was significantly correlated with HAMD score improvements (*r* = 0.74, *p* < 0.001; Figure 3). The HAMD scale changes, right MCC brain image changes, left SMA brain image changes were consistent with normal distribution test.

## Discussion

The present study examined neuroimaging changes with fMRI after taVNS and demonstrated that taVNS was an effective treatment for first-episode, drug-naïve depression. To the best of our knowledge, few studies have explored changes in abnormal neural activity (ReHo) after taVNS treatment in first-episode, drug-naïve MDD patients. The primary finding is that MDD patients exhibited significantly lower ReHo values in the left/right MCC, the left PrCG, the left PoCG, the right CAL, the left SMA, the left PAL, and the right LG after treatments compared with their ReHo values before treatments. Positive correlation was discerned between the decreased ReHo values in the right MCC and in the left SMA and the decreased HAMD scores. This work adds to the body of knowledge about changes in brain functions of MDD patients after taVNS treatments, which may offer researchers insights on the underlying neurological mechanisms of taVNS treatments for MDD.

After taVNS treatments, we detected a significant decrease of ReHo values in bilateral MCC in first-episode, drug-naïve MDD patients. In addition, the right MCC decreased ReHo after taVNS was significantly positively correlated with the decreased of HAMD scores. The MCC has been linked to cognitive regulation, negative affect processing, and pain processing (Wong et al., 2019; Brenner et al., 2021). Decreased functional connectivity (FC) between the MCC and other brain areas was attributed to decreased depression after antidepressant medication (Tan et al., 2020). The initial abnormal increase in resting-state FC from the visual cortex to the cingulate cortex was diminished by repetitive transcranial magnetic stimulation (rTMS), which was associated with relief of depressive symptoms (Zhang et al., 2021). Meta-analysis indicated that MDD patients have a higher amplitude of low-frequency fluctuations (ALFF) in the cingulate gyrus (Gong et al., 2020). Another meta-analysis found lower gray matter (GM) volume in the MCC in MDD patients (Wang et al., 2022). Changes in MCC structures and functions have been regarded as important dimensions to predict depression prognosis (Stuke et al., 2016; Cheng et al., 2017). In summary, the MCC is abnormal in functions and structures in MDD patients, suggesting the importance of MCC in the pathogenesis of MDD. In this study, we found that the neural activity status of MCC in MDD patients was decreased by taVNS, and this reduction was associated with improved longitudinal treatment outcomes. Therefore, we speculate that the functional regulation of MCC may be the effective brain mechanism basis of taVNS treatment for MDD patients.

After taVNS treatments, MDD patients had significantly lower ReHo values in the left SMA, implying that the SMA may act as a key hub of taVNS for antidepressant treatment in MDD patients. Researchers stated that motor movements, motor action sequencing and planning, and response inhibition are all controlled by the SMA (Mohebi et al., 2019; Dong et al., 2021). Compared with HCs, MDD patients were reported to have increased ReHo values in the SMA (Liu et al., 2012; Yan et al., 2021). A previous study discovered significantly higher FC in SMA in MDD patients (Pan et al., 2021). Researchers in treatment-resistant MDD have shown that SMA hypometabolism had active response to antidepressant medication (Jiang et al., 2018). Abnormal GM volume and GM density were found in SMA in MDD patients (Cheng et al., 2010; Jiang et al., 2018) and SMA can predict ECT treatment response in MDD (Jiang et al., 2018). The SMA is abnormal in functions, structures and metabolism in MDD patients, suggesting the importance of SMA in the pathogenesis of MDD. Previous studies (Liu et al., 2012; Yan et al., 2021) showed that the ReHo values of SMA in MDD patients were higher than those in HCs. However, in this study, taVNS significantly decreased the ReHo values of SMA in MDD patients, and the depressive symptoms were significantly relieved after 4 weeks, indicating that taVNS is an effective neuromodulation method for the treatment of MDD. In addition, decreased ReHo values in the left SMA after taVNS were significantly positively correlated with the decreased HAMD scores, further demonstrating the effectiveness of taVNS in the treatment of MDD after regulating SMA brain activity. Combined with these previous studies and our study, we speculate that SMA is expected to be one of the target brain regions for the treatment of MDD in the future.

Our findings indicated that the ReHo values of the left PrCG, PoCG, and PCL in first-episode MDD patients significantly decreased after taVNS treatments. The PrCG, PoCG, and PCL belong to the sensorimotor cortex and are engaged in cognitive functions (Wang et al., 2015), disorganized behavior (Dey et al., 2021), emotional processing (Li et al., 2016), and no-planning impulsivity (Zhang R. et al., 2020). Some recent studies have shown that the ReHo and ALFF values of PrCG and PoCG were higher than those of HCs in MDD patients (Xia et al., 2019; Liu et al., 2021; Yan et al., 2021). Another study found that rTMS reduces the activity of C fibers in the left PrCG and PoCG (Tamura et al., 2004). Previous neuroimaging investigations revealed increased FC between PrCG and other brain regions in MDD patients compared to HCs (Shen et al., 2015). Researchers showed that regional characteristics of neuronal activity in the PoCG was linked to depression severity (Tadayonnejad et al., 2015). In first-episode MDD patients, researchers discovered greater local efficiency of the right PCL when compared to HCs (Chen et al., 2022). Furthermore, stronger PCL-amygdala resting-state FC has been reported in mood disorders with suicidal behavior (Zhang R. et al., 2020). After cognitive behavioral therapy, FC between the PCL and other brain regions decreased in MDD patients (Pantazatos et al., 2020). Some neuroimaging investigations have demonstrated abnormal brain surface area and GM volume (Grieve et al., 2013; Schmaal et al., 2017), cortical thickness (Papmeyer et al., 2015; Bos et al., 2018), and GM density (Jiang et al., 2018) of PrCG, PoCG, and PCL in MDD patients. Taken together, these findings suggest that functional and structural impairments of PrCG, PoCG, and PCL in MDD patients may play an important role in the pathophysiology of MDD. Consistently, taVNS treatment regulated neural activity in these brain regions, and greatly relieved depressive symptoms in MDD patients in our study. taVNS may be involved in regulating cognitive functions and emotional processing in MDD patients, providing novel evidence for the importance of the abovementioned brain regions in regulating the underlying neural circuits and possibility of being the target brain regions in the future therapy

Decreased ReHo values were found in right LG and CAL in MDD patients after taVNS. The LG and CAL belong to the visual cortex and are involved in facial expression and emotion processing (Dima et al., 2018; Sun et al., 2018), and higher levels of visual processing (Fan et al., 2021). Researchers discovered that MDD patients had significantly higher ReHo in LG and CAL than HCs (Shen et al., 2017; Geng et al., 2019). The particular dynamic ALFF change for MDD was discovered in the LG and CAL (Peirce, 2015). Regional cerebral blood flow in the right LG and CAL gyrus was significantly reduced in depression patients treated with ECT as compared to medication (Shi et al., 2022). Patients had a lower functional connection degree (FCD) in the LG after rTMS than previously (Zheng et al., 2020). In veterans with MDD, a prior study found a link between depressed symptoms and increased FC between the CAL and the basolateral amygdala (McGlade et al., 2020). In addition, researchers discovered that baseline functional stability in the CAL could predict clinical symptoms improvement in depressed patients (Li et al., 2021). Some neuroimaging investigations have demonstrated abnormal GM volume (Jung et al., 2014; Zhang Y. et al., 2020), cortical thickness (Suh et al., 2019; Lee et al., 2021) of LG and CAL in MDD patients. An earlier study found a link between visual salience and depression symptoms (Miller et al., 2015). All of these findings highlight the importance of LG and CAL in the neuropathology of MDD, and abnormalities in the function, structure, and cerebral blood flow of LG and CAL may lead to MDD. Changed ReHo values of the LG and CAL in MDD patients was found after taVNS treatment, which can be speculated that the functional regulation of the LG and CAL might be the effective brain mechanism basis of taVNS treatment for MDD patients.

Based on the previous research on brain functions and structures and in combination with our study, we speculate that abnormal functional activation and structure of these brain regions may be closely related to MDD, but the relationship between structure and function is still unclear and needs to be investigated in the future. Besides, we speculate that the taVNS may relieve the emotional and cognitive symptoms of MDD patients by regulating the abnormal neural activities of these brain regions (MCC, PrCG, PoCG, CAL, SMA, PAL, and LG). These brain regions may be effective functional targets for taVNS treatments of MDD patients. This study has advanced our understanding of therapeutic mechanisms and contributed to the development of therapeutic targets for MDD.

## Limitations and strengths

Some limitations of our study should be mentioned. First, because of the limited number of subjects in this study, additional investigations with bigger sample sizes will further validate the statistical power of the findings. Second, no HCs were recruited to be compared with the MDD patients in the brain function at baseline. Third, the current functional results were simply a preliminary investigation of the influence of taVNS transient changes in MDD patients. More functional studies are needed to confirm whether long-term taVNS treatments could improve effectiveness of treatment in MDD patients. Fourth, the results may be influenced by an unbalanced sex-matched cohort (5: 15). Despite the fact that we screened a large number of possible patients (both male and female), only a tiny percentage of them met the inclusion criteria, and the majority of them were females. Future research should try to avoid the imbalance of sex. Fifth, no patients in the placebo group were recruited. The design of the placebo group is very necessary, and we will include patients in the placebo group in future studies to reveal the brain regions that are sensitive to treatment.

The strengths of our study are also obvious. First, all of the MDD patients were first-episode, drug-naïve, and without any comorbidities. Second, we conducted a longitudinal study which tracked a 4-week follow-up clinical outcome. Third, the taVNS can non-invasively stimulate brain areas in a way similar to VNS in terms of reducing depressive symptoms with surgery free, low cost, and more safety.

In conclusion, taVNS is an effective method for MDD treatment. The ReHo changes in the left/right MCC, the left PrCG, the left PoCG, the right CAL, the left SMA, the left PAL, and the right LG may play an important role in the underlying neural mechanisms of taVNS treatment of MDD. In the future, studies with larger sample sizes and longitudinal cohort matched with HCs are much needed. We hope this work will aid clinicians in diagnosis and decision-making and in turn result in more informed treatments for MDD patients.

## Data availability statement

The raw data supporting the conclusions of this article will be made available by the authors, without undue reservation.

## Ethics statement

The studies involving human participants were reviewed and approved by the Ethics Committee of Guang’anmen Hospital, China Academy of Chinese Medical Sciences. The patients/participants provided their written informed consent to participate in this study.

## Author contributions

SY and ZW: software, data curation, writing—original draft, and writing—review and editing. WY: software, data curation, and review and editing. CH, GZ, PL, and YC: data curation. HZ: review and editing. JF, WL, and JL: writing—review and editing and supervision. All authors contributed to the article and approved the submitted version.

## Footnotes

1
http://rfmri.org/DPARSF
